# Influence of the cognitive aspects of caregivers in the adhesion of treatment in children and adolescents with chronic rheumatic diseases-preliminary data

**DOI:** 10.1186/1546-0096-12-S1-P156

**Published:** 2014-09-17

**Authors:** Lívia Keppeke, Claudio A Len, Vanessa Miotto e Silva, Juliana Molina, Fernanda Lewinsky, Maria Teresa Terreri

**Affiliations:** 1 Pediatrics, UNIFESP, São Paulo, Brazil

## Introduction

The adhesion to treatment is a key factor for an effective treatment. Studies have showed considerable taxes in poor adhesion in children and adolescents with rheumatic diseases, which lead to long-term costs and consequences for the patient, the family, and society. Psychological conditions and familial support have demonstrated to be a strong influence in the adhesion to treatment.

## Objectives

To study the psychocognitive and social aspects of the caregivers of patients with rheumatic diseases and evaluate its correlation with adhesion to treatment.

## Methods

42 caregivers of patients followed in our outpatient pediatric rheumatology clinic participated in this study, classified according to good or bad adhesion to treatment, according to the Morisky Green drug adhesion test. The sample was selected consecutively. We used a standard questionnaire to verify the socioeconomic level, the Family Apgar scale to verify family functioning, The Wechsler Adult Intelligence Scale (WAIS) to detect IQ, and clinical and demographic data.

## Results

We observed good adherence in 6% of patients and bad adherence in 31%. In relation to socioeconomic class, 59% (n=17) of adherent patients belonged in the low middle class, against 77% (n=10) of non-adherent patients (p=0.31). The family functioning showed itself to be good in 79% (n=23) of adherent patients and 54% (n=7) of non-adherent patients (p=0.14). In the group of adherent patients, 59% (n=17) of caregivers received help from third parties in the care of patients, while 31% (n=4) of the non-adherent patient group received such help (p=0.18). The total IQ presented an average of 95.2 (SD=7.3) in the adherent patients group and 94.3 (SD=10.9) in the non-adherent patients group (p=0.77).

## Conclusion

We noted a necessity in amplifying our sample size in order to reach statistical significance. Thus far, we observed a trend of higher social economic class in the group of adherent patients, as well as better family functioning and more support from other people in the care of the patients, in comparison with the non-adherent group. The overall average IQ seems similar in both groups. These preliminary results suggest the importance of healthcare teams in conducting reviews seeking global comprehension of the patient to aid in adhesion.

## Disclosure of interest

None declared.

